# Face and Content Validation of the 10-Item Communicative Participation Item Bank General Short Form for Primary Progressive Aphasia: A Cognitive Interviewing Study

**DOI:** 10.1044/2025_AJSLP-25-00085

**Published:** 2025-10-17

**Authors:** Ollie Fegter, Sara Shaunfield, Matthew Bona, Emily Cummings, Angela C. Roberts, Emily Rogalski

**Affiliations:** a Healthy Aging & Alzheimer's Research Care Center, Biological Sciences Division, University of Chicago, IL; b Department of Psychiatry and Behavioral Sciences, Northwestern University Feinberg School of Medicine, Chicago, IL; c Department of Medical Social Sciences, Northwestern University Feinberg School of Medicine, Chicago, IL; d Department of Computer Science, Middlesex College, Western University, London, Ontario, Canada; e School of Communication Sciences and Disorders, Western University, London, Ontario, Canada; f Department of Neurology, Biological Sciences Division, University of Chicago, IL

## Abstract

**Purpose:**

The purpose of this study was to evaluate the face and content validity of the 10-item Communicative Participation Item Bank (CPIB) General Short Form for persons with primary progressive aphasia (PPA) and their communication partners.

**Method:**

Nine dyads, each consisting of a person with PPA and their communication partner, completed semistructured cognitive interviews that assessed the instructions, format, response options, item comprehension, and relevance to their experiences with PPA. Interviews were conducted via videoconference. Closed-ended responses were quantified, and open-ended responses were analyzed via thematic analysis. Summaries were generated for each item, including recommended changes.

**Results:**

Measure instructions and format were clear to all participants (*N* = 18, 100%). Participants demonstrated good comprehension of individual CPIB items, with a majority finding the items clear and relevant to their experiences. Most (*n* = 14, 78%) participants suggested adding a fifth response option (i.e., “somewhat”) to better capture their range of communication experiences. Talking on the phone was the most reported missing communicative participation situation (*n* = 12, 67%), followed by videoconference (*n* = 3, 17%) and e-mail/texting (*n* = 3, 17%).

**Conclusions:**

Initial data support use of the CPIB with persons with PPA and their communication partners for capturing clinically meaningful communication function. Potential modifications for sharpening the relevance, applicability, and sensitivity to longitudinal measurement of change include increasing the number of response levels, adding an item on communicative participation via phone/videochat, and using proctored administration for persons with PPA.

**Supplemental Material:**

https://doi.org/10.23641/asha.30299941

Primary progressive aphasia (PPA) is a clinical neurodegenerative dementia syndrome characterized by insidious onset and progressive decline in comprehension and expressive language skills, such as grammar and word finding, with relative sparing of other cognitive domains (Gorno-Tempini et al., 2004, 2011; Mesulam, 2001; Rogalski & Mesulam, 2009). PPA is pathologically heterogeneous and can be caused by Alzheimer's disease or frontotemporal lobar degeneration (Mesulam et al., 2021; Spinelli et al., 2017). PPA is commonly classified into three subtypes based on predominant clinical features: agrammatic, semantic, and logopenic variants (Gorno-Tempini et al., 2004, 2011; Spinelli et al., 2017). The logopenic variant presents with word finding and phonological difficulties with relatively spared semantic and syntactic abilities, the semantic variant presents with single-word comprehension difficulty and relative sparing of syntactic skills, and the agrammatic variant presents with syntactic impairments with relative sparing of word comprehension abilities (Gorno-Tempini et al., 2004, 2011; Spinelli et al., 2017). These subtypes correspond to distinct patterns of brain atrophy (Rogalski, Cobia, Harrison, Wieneke, Thompson, et al., 2011; Rogalski, Cobia, Harrison, Wieneke, Weintraub, & Mesulam, 2011; Rogalski et al., 2014).

Persons with PPA (PwPPAs) report feelings of isolation, frustration, and depression due to professional and personal role changes and decreased participation in daily activities (Lo et al., 2022; Medina & Weintraub, 2007; Ruggero et al., 2019). There is some evidence that language impairment may be the most significant contributor to impaired activities of daily living for PwPPAs (Moeller et al., 2021). In the absence of a definitive cure, the primary approach to PPA care is nonpharmacological interventions, typically delivered by a speech-language therapist (Carthery-Goulart et al., 2013; Khayum et al., 2012; Kortte & Rogalski, 2013; Taylor-Rubin et al., 2021; Volkmer et al., 2020). As a neurodegenerative condition, the goals of speech therapy for PPA should be centered around maintaining and maximizing communication abilities and quality of life rather than restoring lost language skills (Farrajota et al., 2012; Khayum et al., 2012; Kortte & Rogalski, 2013; Rogalski & Khayum, 2018; Taylor-Rubin et al., 2021; Volkmer et al., 2020).

Communication is defined as exchanging knowledge, information, or feelings in everyday situations through speaking, listening, reading, writing, or nonverbal forms (Hidecker, 2010). Communicative participation is defined as the extent to which individuals use communication to engage in their social roles across environments and social contexts (Davies, 2020). Several frameworks, such as the International Classification of Functioning, Disability and Health (World Health Organization, 2001), Living With Aphasia: Framework for Outcome Measurement (Kagan, 2011), and Family of Participation-Related Constructs (Prinsloo et al., 2024), emphasize the importance of communicative participation as a central focus of intervention to enhance the quality of life for individuals with aphasia. Targeting communicative participation prioritizes valued life situations and personal goals to ensure that intervention leads to meaningful changes in daily life (Baylor & Darling-White, 2020). Thus, accurate measurement of communicative participation across the disease course in PPA is essential for understanding disease impact, developing care plans, and assessing intervention efficacy (Henry & Grasso, 2018; Roberts et al., 2022; Rogalski et al., 2025).

By incorporating patient-reported outcome measures (PROMs), clinicians and researchers can ensure that interventions align with the communication experiences of individuals and reflect meaningful improvements in their daily communication activities (Basch, 2017). PROMs have the potential to support evidence-based practices in speech-language therapy by providing measures of subjective constructs, such as communicative participation, that can be tracked over time (Cohen & Hula, 2020). Cohen, Harnish, et al. (2021) provided guidance for using PROMs in speech-language therapies for individuals with cognitive-communication disorders. This included choosing reliable measures, assessing trends over time, and using PROMs to complement standardized assessments of cognition and language abilities. Others have suggested that PROMs can support clinician–patient communication (Greenhalgh et al., 2018) and facilitate individuals with cognitive-communication disorders to participate in health care decisions (Kramer & Schwartz, 2017).

One measure that captures the communicative participation experiences of individuals with communication disorders, including aphasia, is the Communicative Participation Item Bank (CPIB; Baylor et al., 2009, 2013; Yorkston et al., 2008). The CPIB was developed as a condition-agnostic communicative participation self-report measure for research and clinical practice (Baylor et al., 2009). It was designed to assess communicative participation restrictions resulting from motor speech, voice, language, and cognitive-communication disorders (Yorkston et al., 2008). The initial item bank was generated through literature review and expert input and then refined via cognitive interviews (133 items) and Rasch analysis (94 items) in a laryngeal dystonia (neurologic voice disorder) sample (Baylor et al., 2009; Yorkston et al., 2008). To improve generalizability to other communication disorders, the item bank underwent additional item response theory (IRT) analysis using a mixed sample of individuals with multiple sclerosis, Parkinson's disease, amyotrophic lateral sclerosis, and head and neck cancer (Baylor et al., 2013). This resulted in an item bank of 46 items that can be delivered via computerized adaptive testing (CAT). From these 46 items, a 10-item general short-form version of the CPIB was generated and validated in the same mixed sample (Baylor et al., 2013). The 10-item CPIB General Short Form asks respondents to rate how their condition interferes with various daily activities, such as “asking questions in a conversation,” “communicating in a small group of people,” and “giving someone detailed information.” Responses are anchored on a 4-point Likert scale, ranging from *not at all* to *very much*. The 10-item general short-form CPIB is typically administered in a self-paced, paper-and-pencil format. Both raw and *T* scores (theoretical mean of 50 and standard deviation of 10) are available, with higher scores indicating more favorable communicative participation.

The 10-item CPIB General Short Form has been validated in various populations, including individuals with laryngeal dystonia (Yiu et al., 2021), stroke aphasia (Baylor et al., 2017), and head and neck cancer (Baylor et al., 2013). The CPIB has been used to assess response to interventions in individuals with Parkinson's disease (Bryans et al., 2021), voice disorders (Baylor et al., 2021), and stroke aphasia (Northcott et al., 2015) and to examine the relationship between physical symptoms (e.g., fatigue) and communicative participation in multiple sclerosis (Baylor et al., 2010, 2013; Yorkston et al., 2014). Additionally, it has been used to assess longitudinal decline in amyotrophic lateral sclerosis (Sixt Börjesson et al., 2021), Huntington's disease (Carlozzi et al., 2021), and primary progressive apraxia of speech with and without aphasia (Utianski et al., 2021).

The CPIB was developed as a disorder-agnostic measure (Baylor et al., 2009, 2013; Yorkston et al., 2008), and its application to individuals with PPA has been limited (Roberts et al., 2022; Rogalski et al., 2025; Utianski et al., 2020, 2021, 2023). No study has yet examined its psychometric properties in a PPA-specific population. Given the distinct and progressive language profiles of PPA, evaluating the CPIB's face and content validity in this population is necessary to ensure it reflects the real-world communicative participation restrictions experienced by individuals living with PPA. To fill gaps in the existing CPIB validations studies, the purpose of this study was to evaluate the face and content validity of the 10-item general short-form CPIB in PwPPAs and their communication partners (CPs).

## Method

### Participants

Nine dyads consisting of a PwPPA (*n* = 9) and their CP (*n* = 9) were enrolled in this study. Recommended sample size for cognitive interviews ranges from five to 15 participants per item (Ryan et al., 2012; Turner-Bowker et al., 2018). Our sample of nine dyads (i.e., 18 participants) accounts for potential variability in PPA subtypes and severity, allowing us to capture a range of perspectives. Dyads were eligible if both the PwPPA and their CP were willing and able to participate in the cognitive interviews. Six dyads (“familiar dyads”) had previously completed the CPIB as part of the Communication Bridge–2 randomized controlled trial (NCT03371706; Roberts et al., 2022; Rogalski et al., 2025). The average time between their prior CPIB completion and this study was 10.83 months (*SD* = 4.36, range: 4–16 months). Three dyads (“naïve dyads”) unfamiliar with the CPIB were recruited from the Communication Bridge–3 clinical trial waitlist (NCT06191198). PPA subtype was determined via medical record review of neuropsychological and speech-language assessments, aligned with established guidelines for categorizing PPA subtypes (Gorno-Tempini et al., 2011). We purposively sampled participants to ensure representation from all three primary PPA subtypes (semantic, nonfluent/agrammatic, and logopenic) across both naïve and familiar dyads. See Table 1 for inclusion criteria.

**Table 1.** Inclusion criteria for persons with primary progressive aphasia (PwPPAs) and their communication partners (CPs).

Criteria	PwPPA (*n* = 9)	CP (*n* = 9)
Age	18 years or older	18 years or older
Diagnosis	Meets criteria for PPA, of any subtype, as diagnosed by an experienced clinician (speech-language therapist/pathologist or neurologist) or confirmed via medical record review (by speech-language therapist/pathologist)	N/A
PPA severity	Mild to moderate; determined via the Western Aphasia Battery–Revised Aphasia Quotient (Kertesz, 2007) and the Reading Comprehension subtest of the Boston Diagnostic Aphasia Examination (Goodglass et al., 2001); see caption	N/A
Role	N/A	Spouse or long-term partner of PwPPA, living in the same household or having regular (i.e., daily) extended contact with the PwPPA
Hearing and vision	Adequate (aided or unaided, by self-report) to participate in cognitive interviews	
Reading abilities	Adequate to complete the CPIB (with or without supports)	
Primary language	English primarily used in daily communication activities	
Technology ability	Able to use videoconference (with or without training/assistance)	
Consent	Able to provide consent to study participation (assessed via consent comprehension evaluation)	
Medical conditions	No concurrent serious medical, psychiatric, or neurologic illness (other than PPA) at the time of enrollment	

*Note.* For familiar PwPPAs, the most recent WAB-R AQ score on file was used, and 75 was used as the severity cutoff score. For naive PwPPAs, a speech-language therapist administered the WAB-R AQ, and a severity cutoff score of 80 was used. There is evidence of strong reliability for telehealth administration of the WAB-R in PwPPAs (Rao et al., 2022). The cutoff severity score was increased after the first six interviews with familiar participants based on observed difficulties with interview participation for participants with more severe language difficulties. This adjustment to a higher cutoff score allowed for increased participation in the cognitive interviews while still including individuals with a range of language impairment profiles. Three interviews were conducted with the higher cutoff score of 80, all with naive participants. The Boston Diagnostic Aphasia Examination (BDAE) Reading Comprehension Sentences and Paragraphs subtest was used to ensure adequate reading abilities for completing the study protocol. As PwPPAs who had completed the CPIB as part of the Communication Bridge–2 trial had previously demonstrated adequate reading abilities, only persons with PPA in the naive dyads completed the BDAE Reading Comprehension subtest before enrollment. Participants were required to correctly answer eight of 10 reading comprehension questions to be eligible for enrollment. PPA = primary progressive aphasia; N/A = not applicable; CPIB = Communicative Participation Item Bank; WAB-R = Western Aphasia Battery–Revised; AQ = Aphasia Quotient.

### Procedure

This study was approved by the institutional review boards at both Northwestern University and the University of Chicago. All study materials were developed in consultation with two licensed speech-language therapists to ensure they were accessible and appropriate for individuals with aphasia. All evaluations and interviews were conducted via videoconference. Visual supports were used throughout the screening, consent, and interview process to facilitate comprehending and responding for PwPPAs.

Participants were first contacted via phone and e-mail to gauge interest. If interested, dyads completed a screening call with a trained research staff member to collect demographic information, confirm eligibility, and complete the informed consent process. Understanding of study procedures and consent processes was confirmed for individuals with PPA through a short comprehension assessment administered at the time of consent. If eligible after the initial screening call, naïve dyads completed an additional screening call with a speech-language pathologist to review medical records, confirm PPA diagnosis, and complete a severity screening (see Table 1).

After enrollment, dyads were sent the CPIB questionnaires via mail in a sealed envelope and instructed not to look at the questionnaire until their scheduled interview. Interviews were conducted with familiar dyads first, followed by naïve dyads, to explore potential differences in responses based on CPIB familiarity. For each dyad, the PwPPA interview was scheduled before the CP interview, which was scheduled on a separate day. When requested by the PwPPA, CPs were present during interviews to provide communication and practical support (Schaffer & Henry, 2021). In these cases, interviewers instructed PwPPAs to respond first, with CPs assisting only if needed. Whenever feasible, interviews were conducted with CPs out of earshot to minimize response bias. Participants independently completed the CPIB at the beginning of the call before the semistructured interview described below. If PwPPAs struggled to respond to open-ended questions (e.g., word-finding difficulty), interviewers adapted the interview protocol by asking yes/no questions to assess participants' understanding and gather more specific feedback. Participants returned the questionnaires via mail upon interview completion.

Interviews were recorded using a built-in video recording feature, as well as using an external audio recorder to ensure high-quality transcription. Interviewers took detailed field notes during the interview, which were subsequently entered into Excel. Audio recordings were transcribed verbatim and de-identified prior to analysis.

### Materials

#### CPIB

As described above, the 10-item CPIB General Short Form was used for this study (Baylor et al., 2013). For PwPPAs, a modified format was used to facilitate responding and minimize comprehension difficulties (i.e., one interview question and corresponding CPIB item at a time shown via PowerPoint on a shared Zoom screen; see Supplemental Material S1); no changes were made to item content. This is consistent with the format used as part of the Communication Bridge–2 trial (Roberts et al., 2022; Rogalski et al., 2025). CP forms followed the original format but asked individuals to respond to items in reference to how PPA interferes with their partner's (i.e., the PwPPA) communication activities. As such, with CPs, the CPIB was a proxy measure of communicative participation restriction for the PwPPA. In other words, CPs were not asked to rate their own communicative participation, but rather how PPA affects their partner's communication participation.

#### Cognitive Interview Guides

We employed cognitive interviewing, a qualitative research method designed to explore how respondents understand, interpret, and respond to questionnaires (e.g., instructions, recall period, individual item content; Drennan, 2003; Egger-Rainer, 2019; Peterson et al., 2017). Cognitive interviewing enables the identification of any potential issues with a measure's clarity, relevance, and appropriateness for the target population, ensuring that the items accurately capture the intended constructs from the respondents' perspective (Beatty & Willis, 2007; Willis, 2004). Our cognitive interviewing protocol was informed by Willis' methodology (Willis, 2004) and designed to assess participants' comprehension of individual items and their response processes. Through semistructured interviews, participants evaluated the measure's instructions, format, response options, item clarity, and relevance to their experiences with PPA. Open-ended questions then encouraged participants to share additional relevant communication experiences not covered by the measure. See Supplemental Material S2 for cognitive interviewing guides.

### Data Analysis

Interviews were conducted iteratively, with findings from each interview informing subsequent interviews. Responses to closed-ended questions were tabulated to provide an overview of participants' perceptions of measure instructions, questionnaire format, response options, item clarity, and relevance of individual items to experiences with (PwPPA) and observations of (CP) communicative participation in PPA. Responses to open-ended questions were reviewed and summarized to evaluate comprehension of questionnaire instructions, recall period, items, identify any missing communicative participation content, and recommended changes. For open-ended questions, we conducted an inductive thematic analysis approach during which the analysts (O.F., S.S.) and team met regularly to discuss interpretations and resolve any discrepancies.

To evaluate comprehension of measure instructions and individual items, two trained interviewers (S.S. and O.F.) analyzed detailed field notes and transcripts. Comprehension of measure instructions was based on the participant's (a) ability to follow measure instructions/complete the measure independently, (b) comments or questions while reading the measure instructions, and (c) response to the interview prompt “Tell me what the instructions asked you to do.” Acceptable understanding was indicated when participants correctly followed measure instructions or made relevant comments indicating comprehension of what the instructions asked them to do. Evaluations of item comprehension were based on the participant's (a) responses to individual items, (b) comments and questions while responding to individual items, and (c) responses to the interview question, “What did you think about when you answered this question?” Good understanding of an item was demonstrated when participants provided responses that aligned with the item's intended meaning, asked relevant questions, or provided examples of the communicative participation experiences that aligned with the item content and intent. We assessed measure-level understanding (i.e., participants' comprehension of the overall questionnaire intent) by asking: “What were these questions asking you about?” Good understanding was demonstrated when participants identified the measure's focus on communicative participation and could relate the overall measure content to their own experiences or their CP's experiences. Open-ended responses regarding missing content were collected and analyzed for recurring themes.

Summaries of measure instructions, format, and response options, as well as item-level data regarding comprehension, relevance, and missing content, were generated. Next, these summaries were then reviewed by a qualitative and mixed-methods study investigator (S.S.) specializing in PROM development. Any discrepancies in the interpretations were resolved through team discussion (O.F., S.S., E.R., A.C.R.). Exemplary quotes were extracted from transcripts to illustrate findings and justify proposed modifications to the existing measure.

## Results

See Table 2 for demographic and clinical characteristics of the sample.

**Table 2.** Demographic and clinical characteristics of participants.

Variable	PwPPA (*n* = 9)	CP (*n* = 9)	Total (*N* = 18)
Sex			
Female (%)	4 (44.4)	5 (55.6)	9 (50)
Male (%)	5 (55.6)	4 (44.4)	9 (50)
Age, *M* (*SD*)	71.00 (8.49)	70.44 (8.88)	70.72 (8.43)
Race/ethnicity			
White/Non-Hispanic or Latino origin (%)	9 (100)	9 (100)	9 (100)
Highest level of education			
Some college/technical degree/AA (%)	1 (11.1)	1 (11.1)	2 (11.1)
College degree (BA/BS) (%)	2 (22.2)	4 (44.4)	6 (33.3)
Advanced degree (MA/PhD/MD/JD) (%)	6 (66.7)	4 (44.4)	10 (55.6)
Current employment			
Full time (%)	1 (11.1)	3 (33.3)	4 (22.2)
Part time (%)	0 (0)	1 (11.1)	1 (5.6)
Retired (%)	6 (66.7)	5 (55.6)	11 (61.1)
Unemployed (%)	2 (22.2)	0 (0)	2 (11.1)
Primary subtype			
Agrammatic (%)	5 (55.6)	N/A	N/A
Logopenic (%)	2 (22.2)	N/A	N/A
Semantic (%)	2 (22.2)	N/A	N/A
Months since diagnosis (*SD*)	32.11 (16.71)	N/A	N/A
WAB-R AQ (*SD*)	81.77 (5.89)	N/A	N/A
BDAE Reading Comprehension	8.33 (0.58)	N/A	N/A

*Note.* WAB-R AQ scores from most recent WAB-R administration for familiar participants (*M* = 10.83 months, *SD* = 4.36 months) and SLP-administered WAB-R for naive participants. BDAE Reading Comprehension (Sentences and Paragraphs only) scores for naive participants. PwPPA = person with primary progressive aphasia; CP = communication partner; WAB-R = Western Aphasia Battery–Revised; AQ = Aphasia Quotient; BDAE = Boston Diagnostic Aphasia Examination; SLP = speech-language pathologist; AA = Associate of Arts; BA = Bachelor of Arts; BS = Bachelor of Science; MA = Master of Arts; PhD = Doctor of Philosophy; MD = Doctor of Medicine; JD = Juris Doctor.

### CPIB Instruction Wording

All participants (*n* = 9 PwPPAs, *n* = 9 CPs) reported that the CPIB instructions were clear and demonstrated a good understanding of measure instructions. Two PwPPAs had difficulty describing the measure instructions but showed good comprehension when asked yes/no questions to clarify their understanding. Both participants were familiar with the CPIB and enrolled with the lower Western Aphasia Battery–Revised (WAB-R) Aphasia Quotient (AQ) cutoff score of 75. Most participants (*n* = 6, 67% PwPPAs; *n* = 8, 89% CPs) thought about a specific time period when answering the questionnaire. Among those, time periods ranged from average experiences with communicative participation ranging from the past week to the past year. Most (*n* = 5, 56% PwPPAs; *n* = 6, 67% CPs) anchored responses to the past month. No participants reported thinking about specific situations or experiences. All responses were provided in context to “general” communication situations and communication challenges experienced related to PPA.

### CPIB Format and Response Options

All PwPPAs stated the questionnaire format was clear; one (11%) participant suggested adding an instruction about where to mark one's answer on the page. All PwPPAs reported it was helpful for each question to be listed separately (i.e., each question in its own box) and stated that the format of response options (i.e., answer choices presented on lines) was helpful. All were able to complete the measure independently. Two PwPPAs (22%) asked the interviewer where to mark their answer on the page. All CPs reported the measure format was clear.

When asked if the response options reflect their experiences communicating in a range of different situations and environments, five (56%) PwPPAs spontaneously suggested adding an additional response option (e.g., “somewhat”) between “a little” and “quite a bit” to better capture moderate difficulty in their communication experiences. The four participants who did not spontaneously suggest an additional response option were asked if it would be helpful; two (50%) of these participants responded it would be beneficial. Similarly, three (33%) CPs spontaneously suggested adding a middle “somewhat” response option between “a little” and “quite a bit.” Because of the high volume of suggestions for an additional response option, the remaining six were probed as to the usefulness of adding a middle response option. Two (33%) reported it would be beneficial; two (33%) reported it would have been beneficial earlier in the disease course, when communication was less impaired.

### Item-Level Analysis

**Figure 1.**
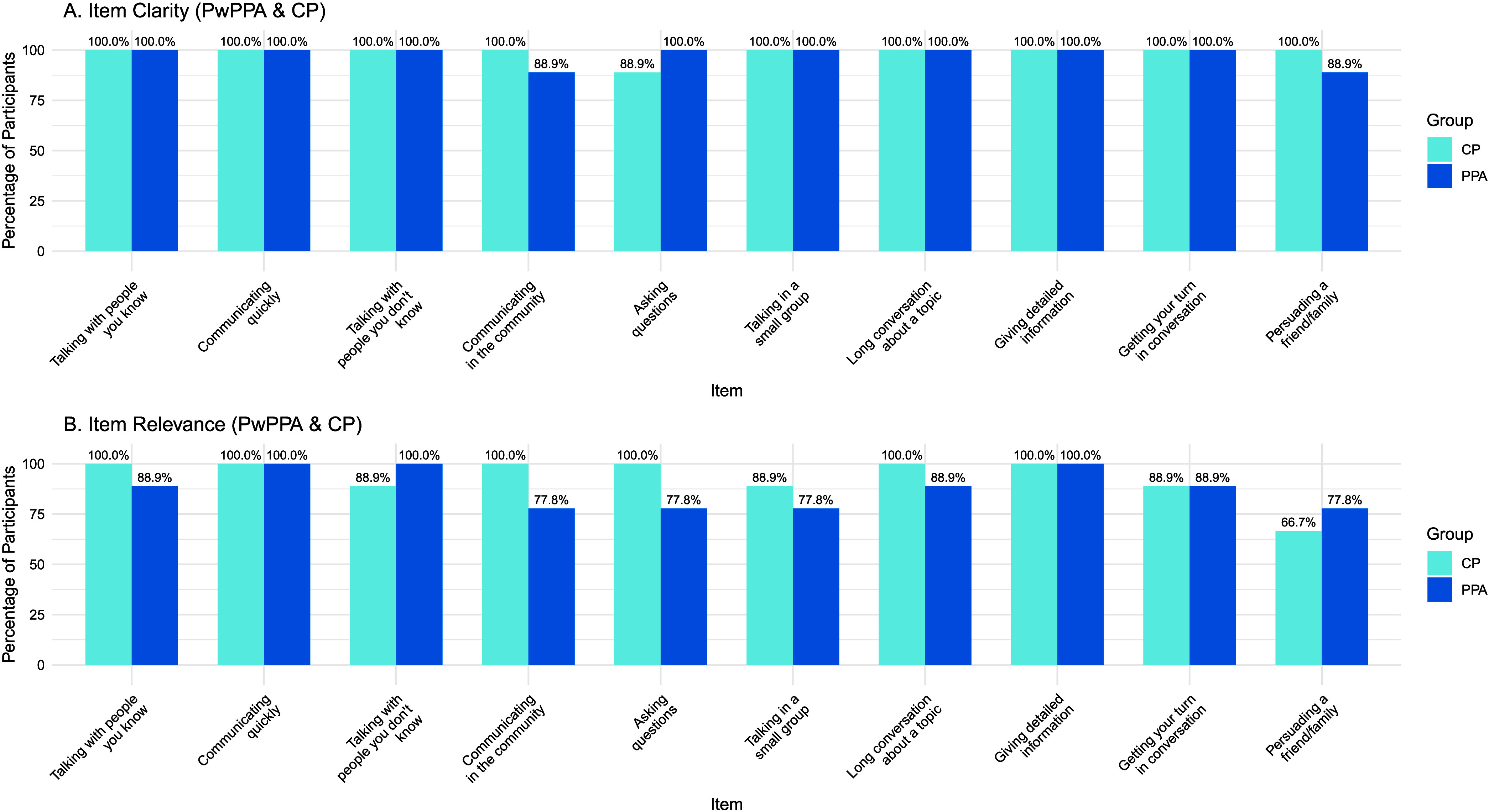
Clarity and relevance ratings. (A) Percentage of participants responding “yes” to the question: “Was the meaning of the question clear?” Clarity ratings were high across all items. (B) Percentage of participants responding “yes” to the question: “Is this question relevant to your/your partner's experiences with PPA?” Relevance ratings varied, with Item 10 (“Trying to persuade a friend or family member to see a different point of view”) reported as relevant for only 72.2% (*n* = 13) of participants. PwPPA = person with primary progressive aphasia; CP = communication partner.

Table 3 provides the CPIB questions by item. For nine of the 10 items, participants reported the items as clear (*N* = 18, range: 94.4%–100%). All CPIB items, except Item 10 (“Trying to persuade a friend or family member to see a different point of view”; 72.2%), were rated as highly relevant to participants' experiences with PPA. Three PwPPAs (33%) and five CPs (56%) indicated that “persuading others to see a different point of view” is either an uncommon situation or not something they engaged in even prior to their PPA diagnosis. For individual item relevance and clarity ratings, see Figure 1.

**Table 3.** Comprehension and participant recommendations for individual items.

Item	Does your/your partner's condition interfere with …		Comprehension summary	Comprehension illustrative quotes	Participant recommended changes
1	Talking with people you know?	PwPPA	All (*n* = 9, 100%) showed good understanding with relevant examples	“The people that I work with. It's easier to speak with, and what have you, than others.”	None
		CP	All (*n* = 9, 100%) showed good understanding with relevant examples	“We have a group of friends that we're getting comfortable with. And then there's my daughters. So, I was going back and forth between those two types of conversations.”	Clarify know well vs. know casually (*n* = 1, 11%)
2	Communicating when you need to say something quickly?	PwPPA	All (*n* = 9, 100%) showed good understanding with relevant examples; 1 (11%) required prompting with yes/no questions due to word-finding difficulties	“I have to slow down. And so, it puts a pressure to me … the condition would make me stop to have to wait.”	None
		CP	All (*n* = 9, 100%) showed good understanding with relevant examples	“Like in an appointment where someone asks him something and he needs to, you know, where we have to kind of move things along, right? He just can't.”	None
3	Talking with people you do NOT know?	PwPPA	Most (*n* = 7, 78%) showed good understanding with relevant examples; 1 (11%) did not provide relevant answer and was unable to be redirected; 1 (11%) was unable to answer even after prompting	“It is difficult talking to people you don't know, and someone that might not know our problems.”	Replace “interfere” with an easier word (*n* = 1, 11%); clarify if talking to another PwPPA (*n* = 1, 11%)
		CP	All (*n* = 9, 100%) showed good understanding with relevant examples; 1 (11%) noted they guessed because they are not around their partner in this situation	“A stranger asking for directions.”	None
4	Communicating when you are out in the community (e.g., errands, appointments)?	PwPPA	All (*n* = 9, 100%) showed good understanding with relevant examples	“Going to the grocery store and I have to ask something where I can't find it or the pharmacy. But before I have to ask them, I could practice in my mind.”	Shorten question (*n* = 1, 11%); add ordering from menu (*n* = 1, 11%); add shopping (*n* = 1, 11%)
		CP	All (*n* = 9, 100%) showed good understanding with relevant examples; 3 (33%) noted they guessed because they are not around their partners in this situation	“I thought about her going grocery shopping or to doctor's appointments, returning something to the store, that kind of thing.”	Add shopping (*n* = 1, 11%); add church (*n* = 1, 11%); add community events (*n* = 1, 11%)
5	Asking questions in a conversation?	PwPPA	Most (*n* = 8, 89%) showed good understanding with relevant examples; 1 (11%) unable to provide an answer/describe their thought process even with prompting	“The problem becomes you can get a question, but to get it into the conversation, you're probably about two seconds back.”	Replace “interfere” with an easier word (*n* = 1, 11%)
		CP	All (*n* = 9, 100%) showed good understanding with relevant examples; 2 (22%) reported this is something their partner is no longer able to do	“Asking a question in a group at work on a call or something.”	Add additional context (e.g., planned vs. spontaneous conversation, familiar vs. unfamiliar subject matter; *n* = 1, 11%)
6	Communicating in a small group of people?	PwPPA	Most (*n* = 7, 78%) showed good understanding with relevant examples; 2 (22%) were unable to provide responses even with prompting	“Probably with my daughter and my husband and son-in-law … I don't want to bother them … if it's not interesting I won't say something.”	Replace “interfere” with an easier word (*n* = 1, 11%); clarify small group of people know vs. don't know (*n* = 1, 11%)
		CP	All (*n* = 9, 100%) showed good understanding with relevant examples	“Being in sort of a small group, you know, three or four people around the dinner table.”	Clarify people know vs. don't know (*n* = 1, 11%)
7	Having a long conversation with someone you know about a book, movie, show, or sports event?	PwPPA	Most (*n* = 8, 89%) showed good understanding with relevant examples; 1 (11%) unable to provide an answer/describe their thought process even with prompting	“My friends. You have friends who have kids who played basketball and baseball.”	Shorten question (*n* = 1, 11%); remove “long” (*n* = 1, 11%); change “about a” to “such as” (*n* = 1, 11%); separate book/movie/sports even (*n* = 1, 11%)
		CP	All (*n* = 9, 100%) showed good understanding with relevant examples; 4 (44%) noted this is something their partner is no longer able to do	“Recent experiences with the games leading up to the Super Bowl. We've had a lot of conversations about football games.”	Movies/shows are the same thing (*n* = 1, 11%); add “idea/issue” (*n* = 1, 11%); remove examples (*n* = 1, 11%); add podcast or doctor's appointment (*n* = 1, 11%)
8	Giving someone detailed information?	PwPPA	Most (*n* = 7, 89%) showed good understanding with relevant examples; 1(11%) was unable to provide responses even with prompting; skipped with 1 (11%) due to participant fatigue	“If I have to give details and I know the details it's just difficult to get the words out … like give someone directions.”	Replace “interfere” with a simpler word (*n* = 1, 11%)
		CP	Most (*n* = 8, 89%) showed good understanding with relevant examples; 1 (11%) unable to explain thought process or provide relevant examples	“If you ask for directions to get to our house … he might say, well, you take the bus. And it's like, okay, well, which bus? He'd have to think about that, and then it's like okay, so, what stop do I get off at? And what do I do? He couldn't give detailed information.”	None
9	Getting your turn in a fast-moving conversation?	PwPPA	Most (*n* = 8, 89%) showed good understanding with relevant examples; 1 (11%) unable to provide an answer/describe their thought process even with prompting	“When people are asking things back and forth, they expect that you're going to ask something about something. And if you don't have something, or you know something, but you can't figure out how to get it out, the next guy says, well, we'll get back to you later, you know?”	Replace “interfere” with a simpler word (*n* = 1, 11%); remove “fast moving” for persons with PPA (*n* = 1, 11%)
		CP	All (*n* = 9, 100%) showed good understanding with relevant examples	“We had a group of friends … and there would be four people talking at once and they all understand each other, and she would be pretty quiet. And she would express ‘I wanted to say something, but it was too quick.’”	Combine with Item 6 (*n* = 1, 11%); clarify with people know vs. don't know (*n* = 1, 11%)
10	Trying to persuade a friend or family member to see a different point of view?	PwPPA	Most (*n* = 8, 89%) showed good understanding with relevant examples; 1 (11%) required prompting with yes/no questions due to word-finding difficulties; 1 (11%) unable to demonstrate understanding or provide relevant examples even with prompting	“I don't try to persuade people.”	Replace “interfere” with a simpler word (*n* = 1, 11%), change “family member” to “family” (*n* = 1, 11%); remove this question and replace with talking on the phone (*n* = 1, 11%)
				“If it's a friend, or family members, people don't have the same thoughts, even though it's your mom, or a friend, or something like that.”	
		CP	All (*n* = 9, 100%) showed good understanding with relevant examples; 5 (56%) noted they had to guess because their partner either never did this, or can no longer do this	“I don't know what he has ever tried to persuade anybody about anything … I just don't think this comes up very often.”	Add N/A option (*n* = 1, 11%); change “persuade” to “see a different point of view or perspective” (*n* = 1, 11%)
				“He will still try, sometimes, to bring up a different point, mostly in the political arena. But it is definitely harder for him to get it out.”	

*Note.* Item comprehension was evaluated based on participants' responses, comments, and answers to the interview question, “What did you think about when you answered this question?” Good understanding was shown when responses aligned with the item's intent or participants provided relevant clarifying questions or examples. PwPPA = person with primary progressive aphasia; CP = communication partner. N/A = not applicable.

Comprehension was high across items. For CPs, each item was understood by ≥ 89% of participants, indicating strong item-level comprehension. PwPPAs demonstrated good understanding for most items (item-level comprehension ≥ 89% of participants) with the exception of Item 3 (“Talking with people you do not know”) and Item 6 (“Talking in a small group of people”), where comprehension was slightly lower (item-level comprehension = 78% of participants). This is possibly due to language complexity. For example, one PwPPA (11%) repeatedly suggested that “interfere” was difficult to comprehend and suggested using a simpler word or phrase (e.g., “gets in the away of”). The most common recommended changes were to clarify context (Items 1, 3, 5, 6, and 9) and include additional examples (Items 4 and 7). Comprehension summaries and participant suggestions for individual items are detailed in Table 3.

While comprehension was generally high across items, a minority of participants expressed difficulty responding to select items (*n* ≤ 3, ≤ 17% for each item noted). Participants expressed the most difficulty responding to Item 2 (“Communicating when you need to say something quickly”), Item 4 (“Communicating when you are out in the community; e.g., errands, appointments”), Item 7 (“Having a long conversation with someone you know about a book, movie, show, or sports event”), and Item 10 (“Trying to persuade a friend or family member to see a different point of view”). For reasons given and illustrative quotes, see Table 4.

**Table 4.** Communicative Participation Item Bank items that were difficult for participants to answer.

Item	Does your/your partner's condition interfere with …	PwPPA	CP	PwPPA + CP	Illustrative quotes (Why was this question difficult to answer?)
2	Communicating when you need to say something quickly?	*n* = 1	*n* = 1	*n* = 2	**CP:** “I don't know what's going on in her head … so when she's very quiet in a conversation, I don't know why … I don't know if she wants to say something quickly.”
4	Communicating when you are out in the community (e.g., errands, appointments)?	*n* = 2	*n* = 1	*n* = 3	**PPA:** “Too much … too long.”
					**PPA:** “I didn't see ‘communicating’ … a lot of words.”
					**CP:** “If I'm with him, I'm usually the one talking and I'm surmising about the times I'm not with him.”
7	Having a long conversation with someone you know about a book, movie, show, or sports event?	*n* = 1	*n* = 1	*n* = 2	**PPA:** “Really long.”
					**CP:** “It was difficult to identify what we have had long conversations about.”
10	Trying to persuade a friend or family member to see a different point of view?	*n* = 1	*n* = 2	*n* = 3	**PPA:** “Don't persuade a lot … except for persuading friends to go to a restaurant.”
					**CP:** “I don't think it comes up often … I don't think we are sitting there trying to persuade each other … I think on a question like this, people might have been like that before they had PPA or that's their personality. That one might not be applicable.”
					**CP:** “That's just not her … I can hardly think of any situation where she'd try to persuade somebody.”

*Note.* PwPPA = person with primary progressive aphasia; CP = communication partner.

### Concept Elicitation

Talking on the phone was the most reported missing communication situation (*n* = 12, 67%; six PwPPAs and six CPs). Talking via videoconference (*n* = 3, 17%; two PwPPAs and one CP) and e-mailing or texting (*n* = 3, 17%; one PwPPAs and two CPs) were the next most reported missing communicative participation situations that were challenging for PwPPAs. All missing communicative participation situations are shown in Table 5.

**Table 5.** Reported missing communication situations.

Situation	PwPPA (*n* = 9)	CP (*n* = 9)	Total (*n* = 18)
Talking on the phone^a^	*n* = 6	*n* = 6	*n* = 12
Videoconference	*n* = 2	*n* = 1	*n* = 3
E-mail/texting	*n* = 1	*n* = 2	*n* = 3
Reading out loud	*n* = 1	*n* = 1	*n* = 2
At work	—	*n* = 2	*n* = 2
Talking to oneself	*n* = 1	*n* = 1	*n* = 2
Talking to others with PPA	*n* = 1	—	*n* = 1
Grocery store^a^	*n* = 1	—	*n* = 1
When can't see someone's face	*n* = 1	—	*n* = 1
Remembering names	*n* = 1	—	*n* = 1
Ordering at a restaurant^a^	*n* = 1	—	*n* = 1
Low speech volume	*n* = 1	—	*n* = 1
Understanding detailed information	—	*n* = 1	*n* = 1
Understanding multiple inputs (e.g., driving and music, conversation with 2+ people)	—	*n* = 1	*n* = 1
Emergency situation^a^	—	*n* = 1	*n* = 1
Writing/handwriting	—	*n* = 1	*n* = 1
Navigating websites	—	*n* = 1	*n* = 1

*Note.* PwPPA = person with primary progressive aphasia; CP = communication partner; PPA = primary progressive aphasia.

a While not included in the 10-Item Communicative Participation Item Bank General Short Form, items indicated with an asterisk here are included in the full 46-item set.

### Measure-Level Summary Questions

When asked what the items as a whole were asking about, all participants showed good understanding by referencing their experiences with communicative participation. PwPPAs described how PPA interferes with their conversations and impacts communication in various situations. CPs similarly emphasized their partner's ability to communicate, noting the ways PPA disrupts different forms of communication and influences their day-to-day functioning. When asked if these questions capture their experience with PPA, most PwPPAs (*n* = 6, 67%) said yes. When asked if these questions capture their partner's experience with PPA, most CPs (*n* = 7, 78%) said yes. For both PwPPAs and CPs, those who said no gave examples of missing communication situations (e.g., comprehension, emergency situations, low speech volume).

## Discussion

The purpose of this study was to evaluate the face and content validity of the 10-item CPIB General Short Form in PwPPAs and their CPs. Results demonstrated good comprehension of the CPIB items among both PwPPAs and their CPs, with most participants reporting that most items were clear and relevant to their experiences (PwPPA) or observations (CP). The two most common suggestions for improving the CPIB for PwPPAs were adding a fifth Likert scale response option (e.g., *somewhat*) and including additional communication situations such as talking on the phone.

Most notably, most participants suggested that the existing 4-point Likert scale did not capture the range of their communication experiences. Participants either spontaneously suggested (44%) or agreed when prompted (28%) that a fifth (e.g., *somewhat*) option would more fully reflect the range of their communicative participation by providing a “middle-ground” option to represent moderate impairment. Interestingly, five response options were originally used during development the CPIB item bank: *not at all*, *a little*, *quite a bit*, *a lot*, and *extremely* (Baylor et al., 2009; Yorkston et al., 2008). IRT analyses suggested that participants did not differentiate between the third response option (“quite a bit”) and the fourth response option (“a lot”). As a result, the response categories were recoded to the four-category set, which has been supported in subsequent IRT analyses (Baylor et al., 2013, 2024). While we recognize the strength of the IRT-driven four-response options, our participants suggested a different five-option structure, with *somewhat* positioned between *a little* and *quite a bit* to represent moderate impairment. It remains important to test whether adding a *somewhat* option would function differently in terms of item difficulty and discrimination. Scale development literature suggests that while 7-point Likert scales may be the most sensitive to change over time (Cano et al., 2006; Preston & Colman, 2000; Simms et al., 2019), 5-point Likert scales offer a similar balance of reliability, validity, and sensitivity, while being less cognitively demanding for respondents with cognitive impairment (Chibnall & Tait, 2001; Zainal et al., 2016). As such, a 5-point Likert scale may better accommodate varying levels of communicative participation restrictions and may be of consideration in future CPIB development specific to this population. This is particularly relevant given recent studies that have highlighted the need for modifications to PROMs for aphasia to reduce respondent burden and enhance accessibility (Engelhoven et al., 2022).

Two CPs suggested that some CPIB items may become less applicable as the syndrome progresses. As communication skills are lost, individuals may no longer be able to participate in quick moving conversations, hold detailed conversations, and so forth. Our sample included individuals classified as having mild, mild–moderate, and moderate aphasia, as determined by their WAB-R AQ scores. Disease duration ranged from 1 to 5 years, reflecting significant variability in the stage of disease progression. This variation in disease severity and duration may have contributed to differences in the applicability of CPIB items, particularly for those in later stages of PPA. As suggested by CPs, a potential modification may be to include a “not applicable” response option for individuals who can no longer participate in various communication situations or those who did not participate in a given activity even prior to their PPA diagnosis. Another option is that if the full CPIB item bank was calibrated for PPA, the CAT version could then be used to tailor assessment to each individual, naturally excluding communication situations that were too difficult for the respondent.

Most participants (67%) reported that talking on the phone is an important communication situation that needs to be added to the current questionnaire. Of note, several items related to talking on the phone were removed from the initial 94-item bank due to local dependence: “talking on the phone to schedule an appointment (e.g., dentist, car repair),” “answering the phone,” “leaving a message on someone's phone,” and “making a phone call for household business” (Baylor et al., 2013). Additional items related to phone conversations are still part of the 46-item bank that can be delivered via CAT but are not included on the 10-item general short form: “making a phone call to get information” and “taking a phone message” (Baylor et al., 2013). While it is clear that our participants expressed a preference for the inclusion of a phone item, IRT analyses conducted during CPIB development indicated that including a phone item did not improve measurement precision (Baylor et al., 2009, 2013, 2017). In other words, IRT analysis suggested stronger psychometric properties when a phone item was excluded. If the goal is to develop a short form that optimizes psychometric precision, it may be more beneficial to exclude the phone item to maintain the scale's psychometric properties. In contrast, including a phone item may enhance the face and content validity of the measure for PwPPAs, including a phone-related item could better reflect the real-world communication experiences of individuals in the early-to-moderate stages of PPA, but this would require recalibration and further psychometric evaluation, including IRT analyses, to assess its impact on the instrument's psychometric properties. Another option may be to administer the item bank via CAT to PwPPAs, as phone items are retained on the CAT version.

The two next most reported missing communication situations were e-mail/texting and talking via videoconference; each was reported as missing by three participants. Interestingly, e-mail and texting were not included at any stage of CPIB development, as the CPIB is intended to measure spoken communication and not written communicative participation (Baylor et al., 2009, 2013; Yorkston et al., 2008).

As videoconference is increasingly used in health care settings (Ashwood et al., 2017; Rush et al., 2018), particularly after the COVID-19 pandemic (Balki et al., 2023; Nguyen et al., 2020), it may be important to consider how PwPPAs are able to participate in videoconference-supported communication. One study recently identified over 300 additional items that could be used to make the CPIB item bank more widely applicable (Ter Wal et al., 2023), including the situations recommended here (i.e., phone, e-mail, texting, videoconference). Adding items related to video- and phone-based communication was also suggested during the recent development of the CPIB–Gender Diverse Short Form (CPIB-GD); an item related to phone conversations was retained in the CPIB-GD after IRT analysis (Baylor et al., 2024; Teixeira et al., 2023). Adding these situations could provide a more comprehensive assessment of the communication challenges faced by this population. As these modalities are increasingly used in therapeutic interventions for PwPPAs, including them could also help track progress in therapy. However, these changes would require additional psychometric validation (Suhr, 2006).

Comprehension of individual items was generally good (see Table 3), with participants rating all items as clear and most items as relevant to their experiences with PPA (see Figure 1). Item 10 was least consistently reported as relevant, with both PwPPAs and their CPs reporting that “trying to persuade a friend or family member to see a different point of view” is not a situation that comes up often in their daily life or even something they did before their PPA diagnosis. As such, this communication situation may be less relevant for PwPPAs.

One PwPPA repeatedly suggested that “interfere” was difficult to comprehend and suggested a simpler phrase such as “gets in the way of.” While the sample is small, this is important to consider as “interfere” is the stem to all CPIB items. PwPPAs may have increased difficulty with low frequency of occurrence words (Fraser et al., 2014; Wilson et al., 2010, 2014). Participants with lower WAB-R AQ scores exhibited more difficulty responding to CPIB items and open-ended interview questions but were able to demonstrate comprehension when interview questions were adapted to yes/no questions versus open-ended questions. This is consistent with a validation study of the CPIB done in stroke aphasia, which found that individuals with a WAB-R AQ score greater than 80 showed acceptable comprehension of CPIB items but required occasional assistance, while those with WAB-R AQ scores lower than 50 were often unable to complete the CPIB (Baylor et al., 2017). Given this response variability, item-level responses may need to be interpreted cautiously.

Although yes/no questioning to assess item comprehension is not part of traditional cognitive interviewing protocols, the CPIB administration was adapted in this study to allow for yes/no questions to facilitate participation for those with more significant language impairments. While PROMs are typically completed independently via paper-and-pencil or electronic administration (Gwaltney et al., 2008; Osoba, 2007; Snyder et al., 2012), there is some support for proctored administration to allow respondents to ask questions while completing a measure (Ashley et al., 2013; Gilbert et al., 2015). Our findings suggest that while independent completion of the CPIB may be feasible for those with mild-to-moderate PPA, individuals may benefit from proctored administration to confirm comprehension and facilitate responding.

Comprehension of measure instructions was generally good. Measure instructions ask respondents to think about the *average* day when responding. PwPPAs and their CPs generally considered general experiences, anchoring their responses to time periods ranging from the past week to the past year, with most considering the past month. Given this variability, it may be beneficial to specify an intended timeframe in the measure instructions. Further qualitative work is needed to determine the most appropriate timeframe for communicative participation recall in this population.

For this study, a modified CPIB form was used for PwPPAs. This modified form listed each question in its own box, which PwPPAs reported was helpful, clear, and easy to use. Prior research has suggested it may be beneficial to simplify language and modify response forms for adults with aphasia and other cognitive impairments (Chue et al., 2010; Cohen, Lanzi, & Boulton, 2021). Our results support the feasibility of using a modified CPIB form for PwPPAs. As comparing measure formats was beyond the scope of this study, future work could systematically evaluate different response formats to determine which are most accessible and effective for individuals with PPA and related conditions.

While the sample sizes of both the naïve and familiar dyad groups were small, we did not observe significant differences between the groups in terms of feedback regarding instruction wording, format, response options, item-level analysis, missing content, or measure-level comprehension. This suggests that familiarity with the measure did not substantially influence participant feedback. However, it is possible that familiarity may have influenced ratings in more subtle ways.

To our knowledge, this is the first study to employ a formal cognitive interviewing protocol to evaluate the face and content validity of a self-report measure of communicative participation in individuals with PPA. While adaptations to traditional cognitive interviewing processes were needed (e.g., presenting CPIB items and interview questions on the screen, probing with yes/no questions) to ensure comprehension and facilitate responding, this study demonstrates the feasibility of directly involving PwPPAs and their CPs in measure development and evaluation. Additionally, we were able to include participants of different PPA subtypes and with varying levels of familiarity with the CPIB to provide a more nuanced understanding of how the measure functions across different groups. The results of this study underscore the importance of PROMs, such as the CPIB, in measuring the real-world communicative participation experiences of individuals with PPA. PROMs such as the CPIB may better reflect the day-to-day experiences of PwPPAs and their partners (Utianski et al., 2021). This study highlights the potential of PROMs to offer a more nuanced understanding of disease progression and intervention efficacy, ensuring that care remains aligned with the evolving needs of individuals with PPA.

This study has limitations. While our sample size is consistent with other cognitive interviewing studies and established sample size recommendations (Ryan et al., 2012; Turner-Bowker et al., 2018), as with all qualitative studies, sample sizes may limit the generalizability of our findings. Specifically, this study focused solely on individuals with mild-to-moderate PPA, and results may not apply to individuals with more severe language impairments. While language is the primary domain affected early in the PPA course, as the disease progresses, other cognitive domains can become compromised. In these cases, insight may become impaired (Banks & Weintraub, 2008; Mesulam & Weintraub, 2008), and additional communication supports and collateral report may be needed when administering patient report measures (Frank et al., 2011) to ensure outcomes remain meaningful. During the study, we increased the WAB-R AQ cutoff score from 75 to 80 after the first six interviews with familiar dyads (see Table 1). This adjustment was made to include individuals with milder language difficulties, thereby increasing participation in the interview process. However, this adjustment complicates the interpretation of our results.

## Conclusions

In summary, this study provides evidence that the CPIB is a valid tool for assessing communicative participation in individuals with PPA; however, some modifications may be helpful to enhance its relevance and applicability. Critical next steps would involve piloting modified items and response scales according to the results presented here (e.g., 5-point Likert scale, including an item to assess talking on the phone or via videoconference), followed by a psychometric validation study to assess the modified scale's structure, reliability, sensitivity to change, and item responses. Importantly, future work should include differential item functioning analyses to determine whether CPIB items function similarly in individuals with PPA compared to the original calibration samples, or whether diagnosis-specific calibration is needed. A modified scale should be validated using a larger, more diverse sample of individuals with PPA, including those with more severe language impairments, to ensure its applicability across the full spectrum of PPA severity. Longitudinal research will be important to assess the CPIB's sensitivity to changes in communicative participation over time in individuals with PPA. As a neurodegenerative condition, these studies could provide valuable insights into how communicative participation evolves alongside language impairment and more diffuse cognitive impairment as the disease progresses. Assessing communicative participation is a critical aspect of PPA care, and the CPIB has the potential to be a valuable resource in both research and clinical intervention.

## Ethics Statement

This study was approved by the institutional review boards at both Northwestern University (STU00218386) and the University of Chicago (IRB23-1347).

## Data Availability Statement

The data sets generated and/or analyzed during the current study are not publicly available to protect participant confidentiality but are available from the corresponding author on reasonable request.

## Supplementary Material

10.1044/2025_AJSLP-25-00085SMS1 Supplemental Material S1 PowerPoint slides used to display cognitive interview questions and CPIB items to PwPPA during interviews.

10.1044/2025_AJSLP-25-00085SMS2 Supplemental Material S2 Cognitive interview guides that were used with both persons with PPA and their communication partners.
