# Supplementary material for: Face and Content Validation of the 10-Item Communicative Participation Item Bank General Short Form for Primary Progressive Aphasia: A Cognitive Interviewing Study
Source: Am J Speech Lang Pathol. 2025 Oct 17;34(6):3300–17. doi: 10.1044/2025_AJSLP-25-00085 (PMC12611418; doi:10.1044/2025_AJSLP-25-00085)
Supplement: Supplemental Material S1 [file AJSLP-34-3300-s001.pdf]

What was your answer for this question?

1. Does your condition interfere with talking with people you know?

What did you think about when you answered this question?

1. Does your condition interfere with talking with people you know?

Was the meaning of the question clear?

1. Does your condition interfere with talking with people you know?

What does the phrase

interfere with

mean?

1. Does your condition interfere with talking with people you know?

For this question, how much would your answer need to change to have a meaningful impact on your life?

1. Does your condition interfere with talking with people you know?

Is this question relevant to your experiences with PPA?

1. Does your condition interfere with talking with people you know?

Should we make changes to this question?

1. Does your condition interfere with talking with people you know?

What was your answer for this question?

2. Does your condition interfere with communicating when you need to say something quickly?

What did you think about when you answered this question?

2. Does your condition interfere with communicating when you need to say something quickly?

Was the meaning of the question clear?

2. Does your condition interfere with communicating when you need to say something quickly?

For this question, how much would your answer need to change to have a meaningful impact on your life?

2. Does your condition interfere with communicating when you need to say something quickly?

Is this question relevant to your experiences with PPA?

2. Does your condition interfere with communicating when you need to say something quickly?

Should we make changes to this question?

2. Does your condition interfere with communicating when you need to say something quickly?

What was your answer to this question?

3. Does your condition interfere with talking with people you do NOT know?

What did you think about when you answered this question?

3. Does your condition interfere with talking with people you do NOT know?

Was the meaning of the question clear?

3. Does your condition interfere with talking with people you do NOT know?

For this question, how much would your answer need to change to have a meaningful impact on your life?

3. Does your condition interfere with talking with people you do NOT know?

Is this question relevant to your experiences with PPA?

3. Does your condition interfere with talking with people you do NOT know?

Should we make changes to this question?

3. Does your condition interfere with talking with people you do NOT know?

What was your answer for this question?

4. Does your condition interfere with communicating when you are out in your community (e.g., errands, appointments)?

What did you think about when you answered this question?

4. Does your condition interfere with communicating when you are out in your community (e.g., errands, appointments)?

Was the meaning of the question clear?

4. Does your condition interfere with communicating when you are out in your community (e.g., errands, appointments)?

For this question, how much would your answer need to change to have a meaningful impact on your life?

4. Does your condition interfere with communicating when you are out in your community (e.g., errands, appointments)?

Is this question relevant to your experiences with PPA?

4. Does your condition interfere with communicating when you are out in your community (e.g., errands, appointments)?

Should we make changes to this question?

4. Does your condition interfere with communicating when you are out in your community (e.g., errands, appointments)?

What was your answer for this question?

5. Does your condition interfere with asking questions in a conversation?

What did you think about when you answered this question?

5. Does your condition interfere with asking questions in a conversation?

Was the meaning of the question clear?

5. Does your condition interfere with asking questions in a conversation?

For this question, how much would your answer need to change to have a meaningful impact on your life?

5. Does your condition interfere with asking questions in a conversation?

Is this question relevant to your experiences with PPA?

5. Does your condition interfere with asking questions in a conversation?

Should we make changes to this question?

5. Does your condition interfere with asking questions in a conversation?

What was your answer for this question?

6. Does your condition interfere with communicating in a small group of people?

What did you think about when you answered this question?

6. Does your condition interfere with communicating in a small group of people?

Was the meaning of the question clear?

6. Does your condition interfere with communicating in a small group of people?

For this question, how much would your answer need to change to have a meaningful impact on your life?

6. Does your condition interfere with communicating in a small group of people?

Is this question relevant to your experiences with PPA?

6. Does your condition interfere with communicating in a small group of people?

Should we make changes to this question?

6. Does your condition interfere with communicating in a small group of people?

What was your answer for this question?

7. Does your condition interfere with having a long conversation with someone you know about a book, movie, show, or sports event?

What did you think about when you answered this question?

7. Does your condition interfere with having a long conversation with someone you know about a book, movie, show, or sports event?

Was the meaning of the question clear?

7. Does your condition interfere with having a long conversation with someone you know about a book, movie, show, or sports event?

What does the phrase

long conversation

mean?

7. Does your condition interfere with having a long conversation with someone you know about a book, movie, show, or sports event?

For this question, how much would your answer need to change to have a meaningful impact on your life?

7. Does your condition interfere with having a long conversation with someone you know about a book, movie, show, or sports event?

Is this question relevant to your experiences with PPA?

7. Does your condition interfere with having a long conversation with someone you know about a book, movie, show, or sports event?

Should we make changes to this question?

7. Does your condition interfere with having a long conversation with someone you know about a book, movie, show, or sports event?

What was your answer to this question?

8. Does your condition interfere with giving someone DETAILED information?

What did you think about when you answered this question?

8. Does your condition interfere with giving someone DETAILED information?

Was the meaning of the question clear?

8. Does your condition interfere with giving someone DETAILED information?

What does the word/phrase

detailed information

mean?

8. Does your condition interfere with giving someone DETAILED information?

For this question, how much would your answer need to change to have a meaningful impact on your life?

8. Does your condition interfere with giving someone DETAILED information?

Is this question relevant to your experiences with PPA?

8. Does your condition interfere with giving someone DETAILED information?

Should we make changes to this question?

8. Does your condition interfere with giving someone DETAILED information?

What was your answer to this question?

9. Does your condition interfere with getting your turn in a fast-moving conversation?

What did you think about when you answered this question?

9. Does your condition interfere with getting your turn in a fast-moving conversation?

Was the meaning of the question clear?

9. Does your condition interfere with getting your turn in a fast-moving conversation?

What does the phrase

getting your turn

mean?

9. Does your condition interfere with getting your turn in a fast-moving conversation?

For this question, how much would your answer need to change to have a meaningful impact on your life?

9. Does your condition interfere with getting your turn in a fast-moving conversation?

Is this question relevant to your experiences with PPA?

9. Does your condition interfere with getting your turn in a fast-moving conversation?

Should we make changes to this question?

9. Does your condition interfere with getting your turn in a fast-moving conversation?

What was your answer to this question?

10. Does your condition interfere with trying to persuade a friend or family member to see a different point of view?

What did you think about when you answered this question?

10. Does your condition interfere with trying to persuade a friend or family member to see a different point of view?

Was the meaning of the question clear?

10. Does your condition interfere with trying to persuade a friend or family member to see a different point of view?

What does the phrase

to persuade

mean?

10. Does your condition interfere with trying to persuade a friend or family member to see a different point of view?

For this question, how much would your answer need to change to have a meaningful impact on your life?

10. Does your condition interfere with trying to persuade a friend or family member to see a different point of view?

Is this question relevant to your experiences with PPA?

10. Does your condition interfere with trying to persuade a friend or family member to see a different point of view?

Should we make changes to this question?

10. Does your condition interfere with trying to persuade a friend or family member to see a different point of view?
