# Supplementary material for: Face and Content Validation of the 10-Item Communicative Participation Item Bank General Short Form for Primary Progressive Aphasia: A Cognitive Interviewing Study
Source: Am J Speech Lang Pathol. 2025 Oct 17;34(6):3300–17. doi: 10.1044/2025_AJSLP-25-00085 (PMC12611418; doi:10.1044/2025_AJSLP-25-00085)
Supplement: Supplemental Material S2 [file AJSLP-34-3300-s002.pdf]

CP ID ____________ Date____________

Evaluation of the 10-item Communicative Participation Item Bank (CPIB) for Persons with Primary Progressive Aphasia and their Communication Partners

Cognitive Interview Guide
CP Version 1.2: January 13, 2023

PART I. INTRODUCTION AND REVIEW OF COMMUNICATION PARTICIPATION ITEM BANK (CPIB)-COMMUNICATION PARTNER

Read to Participant:

The purpose of this interview is to get your feedback on a brief questionnaire called the Communication Participation Item Bank (CPIB)—Communication Partner. We're interested in your candid thoughts and opinions, there are no right or wrong answers. The interview is being recorded so that I don't miss any of your comments. I want to emphasize that your comments are strictly confidential. No names will be used in any reports or publications. All names and other identifying information will be removed from the transcripts. Do you have any questions before we begin?

[TURN ON RECORDER; STATE PARTICIPANT ID AND DATE]

[Interviewer: Participant completes the CPIB questionnaire. Keep the recorder ON as s/he completes the instrument to capture any questions the participant may have about how to complete the questionnaire, or any confusion expressed by the participant].

You just completed a questionnaire called the Communication Participation Item Bank (CPIB)—Communication Partner. We would appreciate your feedback regarding this questionnaire to understand whether the questionnaire is clear and is relevant to communication situations you observe in your partner with PPA. Before discussing your thoughts of each of the questions individually, I'd first like to ask you some general questions about the questionnaire overall.

1. Did you think about a specific period of time, when answering these questions? ___ No (0)
___ Yes (1)

If No → **How did you decide how to respond to/answer these questions?**

If Yes → **What period of time did you think about when answering the questionnaire?**

2. Tell me in your own words what you understood to be the instructions?

3. Were the instructions clear to you? ___ No (0)
___ Yes (1)

If No →**Why?**

4. What did you think about the format of the questionnaire? _________________

5. Was the format of the questionnaire clear to you? ___ No (0)
___ Yes (1)

If No → **What was unclear about the format?**

If No →**Do you have any suggestions on how to reformat the questionnaire to make it more clear?**

**[Interviewer: Ask optional probe below to elicit input about questionnaire formatting if not discussed in response to Q4. Take note of all reformatting suggestions provided by the participant].*

Optional probes:

- **What are your thoughts of the layout of each question?** (e.g., first part of question at top and content on different line)

6. These questions ask you to respond using "Not at all, A little, Quite a bit, and Very much". Do these response options reflect the range of experiences you observe when your partner communicates in different situations/environments?

___ No (0)
___ Yes (1)

If No →**Why?**

7. Was it easy to respond using those response options?

___ No (0)

___ Yes (1)

If No →**Why?**

8. Next, I'm going to ask you a series of questions about each of the questions on the questionnaire. [*Interviewer: If participant appears confused when responding to the questionnaire or when discussing specific items (e.g., struggles/asks questions about an item, changes his/her answer after discussing an item) mark the checkbox in Column 1 for that item and take notes on the issue.*]

**Item**<br><br>**[ASK FOR RESPONSE]**	**a. What did you think about when you answered the question?**<br>*(i.e., How did you come to the answer you gave?)*	**b. Was the meaning of the question clear to you?**<br>Yes/No<br>**If no, what was unclear?**<br><br>**c. *Optional:***<br>***What does the word / phrase _____ mean to you as used in this question?**	**DO NOT ASK IF ANSWERED 3 "NOT AT ALL".**<br><br>**d. For this question, how much would your answer need to change for it to have a meaningful impact on your partner's life?** *(e.g., If your score improved 1 point from __ to __, would that be meaningful to your partner's life? Please explain.)*	**e. Is this question relevant to your partner's experiences with PPA?**<br>Yes/No<br>*If No*→**Why?**<br><br>**f. Do you recommend any changes to this question?**<br>Yes/No<br>**If yes, what changes?**	**ASK IF CP RESPONSE DIFFERS FROM PwPPA**<br><br>**g. I see that that you answered X and your partner answered X for to this question. Can think of a reason why you and your partner may have answered differently?**<br>*(Emphasize: No right or wrong answers, we are interested in learning why answers may differ).*
1. Talking with people he/she knows?<br><br>☐	**a.**	**b.**<br><br>**c.** *interfere with*	**d.**	**e.**<br><br>**f.**	CP response: _______<br><br>PwPPA response: _______<br><br>**g.**

Item<br><br>**[ASK FOR RESPONSE]**	**a. What did you think about when you answered the question?**<br>*(i.e., How did you come to the answer you gave?)*	**b. Was the meaning of the question clear to you?**<br>Yes/No<br>**If no, what was unclear?**<br><br>**c.** ***Optional:***<br>***What does the word / phrase _____ mean to you as used in this question?**	**DO NOT ASK IF ANSWERED 3 "NOT AT ALL".**<br><br>**d. For this question, how much would your answer need to change for it to have a meaningful impact on your partner's life?** *(e.g., If your score improved 1 point from __ to __, would that be meaningful to your partner's life? Please explain.)*	**e. Is this question relevant to your partner's experiences with PPA?**<br>Yes/No<br>*If No*→**Why?**<br><br>**f. Do you recommend any changes to this question?**<br>Yes/No<br>**If yes, what changes?**	**ASK IF CP RESPONSE DIFFERS FROM PwPPA**<br><br>**g. I see that that you answered X and your partner answered X for to this question. Can think of a reason why you and your partner may have answered differently?**<br>*(Emphasize: No right or wrong answers, we are interested in learning why answers may differ).*
2. Communicat-ing when he/she needs to say something quickly?<br><br>☐	**a.**	**c.**<br><br>**d.** *N/A*	**e.**	**f.**<br><br>**g.**	CP ________<br>PwPPA ________<br>**h.**
3. Talking with people he/she does NOT know?<br><br>☐	**b.**	**c.**<br><br>**d.** *N/A*	**e.**	**f.**<br><br>**g.**	CP ________<br>PwPPA ________<br>**h.**

Item<br><br>[ASK FOR RESPONSE]	a. What did you think about when you answered the question?<br>*(i.e., How did you come to the answer you gave?)*	b. Was the meaning of the question clear to you?<br>Yes/No<br>**If no**, what was unclear?<br><br>c. *Optional:*<br>*What does the word / phrase _____ mean to you as used in this question?	DO NOT ASK IF ANSWERED 3 "NOT AT ALL".<br><br>d. For this question, how much would your answer need to change for it to have a meaningful impact on your partner's life? *(e.g., If your score improved 1 point from __ to __, would that be meaningful to your partner's life? Please explain.)*	e. Is this question relevant to your partner's experiences with PPA?<br>Yes/No<br>*If No*→**Why?**<br><br>f. Do you recommend any changes to this question?<br>Yes/No<br>If yes, what changes?	ASK IF CP RESPONSE DIFFERS FROM PwPPA<br><br>g. I see that that you answered X and your partner answered X for to this question. Can think of a reason why you and your partner may have answered differently?<br>*(Emphasize: No right or wrong answers, we are interested in learning why answers may differ).*
4. Communicat-ing when he/she is out in the community (e.g., errands, appointments)?<br>☐	b.	c.<br><br>d. *N/A*	e.	f.<br><br>g.	CP ________<br>PwPPA ________<br>h.
5. Asking questions in a conversation?<br>☐	b.	c.<br><br>d. *N/A*	e.	f.<br><br>g.	CP ________<br>PwPPA ________<br>h.

Item<br><br>**[ASK FOR RESPONSE]**	**a. What did you think about when you answered the question?**<br>*(i.e., How did you come to the answer you gave?)*	**b. Was the meaning of the question clear to you?**<br>Yes/No<br>**If no, what was unclear?**<br><br>**c. *Optional:***<br>***What does the word / phrase _____ mean to you as used in this question?**	**DO NOT ASK IF ANSWERED 3 "NOT AT ALL".**<br><br>**d. For this question, how much would your answer need to change for it to have a meaningful impact on your partner's life?** *(e.g., If your score improved 1 point from __ to __, would that be meaningful to your partner's life? Please explain.)*	**e. Is this question relevant to your partner's experiences with PPA?**<br>Yes/No<br>*If No*→**Why?**<br><br>**f. Do you recommend any changes to this question?**<br>Yes/No<br>**If yes, what changes?**	**ASK IF CP RESPONSE DIFFERS FROM PwPPA**<br><br>**g. I see that that you answered X and your partner answered X for to this question. Can think of a reason why you and your partner may have answered differently?**<br>*(Emphasize: No right or wrong answers, we are interested in learning why answers may differ).*
6.Communicat-ing in a small group of people?<br>☐	**b.**	**c.**<br><br>**d.** *N/A*	**e.**	**f.**<br><br>**g.**	CP ________<br>PwPPA ________<br>**h.**
7. Having a long conversation with someone he/she knows about a book, movie, show, or sports event?<br>☐	**b.**	**c.**<br><br>**d.** *long conversation*	**e.**	**f.**<br><br>**g.**	CP ________<br>PwPPA ________<br>**h.**

Item<br><br>[ASK FOR RESPONSE]	a. **What did you think about when you answered the question?**<br>*(i.e., How did you come to the answer you gave?)*	**b. Was the meaning of the question clear to you?**<br>Yes/No<br>**If no, what was unclear?**<br><br>**c. *Optional:*<br>*What does the word / phrase _____ mean to you as used in this question?**	**DO NOT ASK IF ANSWERED 3 "NOT AT ALL".**<br><br>**d. For this question, how much would your answer need to change for it to have a meaningful impact on your partner's life?** *(e.g., If your score improved 1 point from __ to __, would that be meaningful to your partner's life? Please explain.)*	**e. Is this question relevant to your partner's experiences with PPA?**<br>Yes/No<br>*If No*→**Why?**<br><br>**f. Do you recommend any changes to this question?**<br>Yes/No<br>**If yes, what changes?**	**ASK IF CP RESPONSE DIFFERS FROM PwPPA**<br><br>**g. I see that that you answered X and your partner answered X for to this question. Can think of a reason why you and your partner may have answered differently?**<br>*(Emphasize: No right or wrong answers, we are interested in learning why answers may differ).*
8. Giving someone DETAILED information?<br><br>☐	**b.**	**c.**<br><br><br>**d.** *detailed information*	**e.**	**f.**<br><br><br>**g.**	CP ________<br><br>PwPPA ________<br><br>**h.**
9. Getting his/her turn in a fast-moving conversation?<br><br>☐	**b.**	**c.**<br><br><br>**d.** *getting his/her turn*	**e.**	**f.**<br><br><br>**g.**	CP ________<br><br>PwPPA ________<br><br>**h.**

Item<br><br>[ASK FOR RESPONSE]	**a. What did you think about when you answered the question?**<br>*(i.e., How did you come to the answer you gave?)*	**b. Was the meaning of the question clear to you?**<br>Yes/No<br>**If no, what was unclear?**<br><br>**c.** ***Optional:*** ***What does the word / phrase _____ mean to you as used in this question?**	**DO NOT ASK IF ANSWERED 3 "NOT AT ALL".**<br><br>**d. For this question, how much would your answer need to change for it to have a meaningful impact on your partner's life?** *(e.g., If your score improved 1 point from __ to __, would that be meaningful to your partner's life? Please explain.)*	**e. Is this question relevant to your partner's experiences with PPA?**<br>Yes/No<br>*If No*→**Why?**<br><br>**f. Do you recommend any changes to this question?**<br>Yes/No<br>**If yes, what changes?**	**ASK IF CP RESPONSE DIFFERS FROM PwPPA**<br><br>**g. I see that that you answered X and your partner answered X for to this question. Can think of a reason why you and your partner may have answered differently?**<br>*(Emphasize: No right or wrong answers, we are interested in learning why answers may differ).*
10. Trying to persuade a friend or family member to see a different point of view?<br><br>☐	**b.**	**c.**<br><br>**d.** *to persuade*	**e.**	**f.**<br><br>**g.**	CP ________<br><br>PwPPA ________<br><br>**h.**

PART II. SUMMARY QUESTIONS

Next, I'd like to ask you a few more questions about your thoughts on the questionnaire overall.

1. **In general, what do you think the questions you answered are asking about?**

2. **Please take a moment to look over the questions again. Do these questions, in your opinion, capture your partner's experiences with PPA as it relates to his/her ability to communicate in the most important situations and environments?**

   ___ No (0)

   ___ Yes (1)

   If No →**Why?**

3. **Which questions, if any, were difficult for you to answer?** *For each question identified, ask:* **Can you tell me what about the question made it difficult to answer?** [*Interviewer takes note of questions and reasons.*]

4. **Before we wrap up, I'd like you to take a moment and look over the questions again. Can you tell me about any other important situations/environments in which your partner has trouble communicating that are missing from the questionnaire?** [*Interviewer takes note of questions/situations below and probes for details and examples of each]*

___ No (0)

___ Yes (1)

4a. List all missing communication experiences/situations provided by participant	***4b. Rating***
____	____________
____	____________
____	____________
____	____________
____	____________
____	____________
____	____________
____	____________

4b. *[Interviewer ask if completed Q4a]:* **Next, I would like you to think about how important each of these communication experiences/situations are to your partner's ability to participate in his/her life. On a 0-10 scale, please rate each of these communication experiences/situations to your partner's ability to participate in his/her life. For the rating <u>0 = Not at all important</u> and <u>10 = Extremely important</u>.** *[Interviewer: Read each of the experiences/ situations listed under 4a and ask them to rate it from 0-10. Indicate the rating next to the concern in Column **4b** above. Encourage PT to select a whole number]*

4c. *For the situations/experiences rated the highest (typically 1-2 concerns, but possibly more), ask the participant:* **You rated _______ as one of the most important communication situations/experiences to your partner's ability to participate in his/her life. Why do you believe that situation/experience is the most important?**

5. **Is there anything else that you would like to suggest that would help us to improve these questions or the questionnaire for future use?**

PART III. CLOSING

Thank you for your sharing your feedback and experiences with me today. Your input is very important in helping us to evaluate the appropriateness of this questionnaire for individuals with PPA and their communication partners.

[TURN OFF RECORDER].

[Mailed Gift Card] **Before I let you go; I need to get your mailing address so we can send you your $50 gift card. Would you like us to send the gift card to the same address we mailed the questionnaires?** [*If different address, interviewer writes name and mailing address below.*] **When you receive your $50 gift card, please sign and date the enclosed form and send it back to me in the stamped and addressed envelope provided. I need the form to process the paperwork.**

Name:

Mailing Address:

[Virtual Gift Card] **We will send you a $50 virtual gift card. What email address would you like the gift card to be sent to? You will receive an email within 72 hours from Ollie Fegter, part of the Communication Bridge study team, with instructions on how to activate and access your virtual gift card.**

>>For interviewer: Even if you have an email address on file and participant says "the same email that you sent the consent to," be sure to repeat the email address back to them to confirm.

Email Address:

Evaluation of the 10-item Communicative Participation Item Bank (CPIB) for Persons with Primary Progressive Aphasia and their Communication Partners

Cognitive Interview Guide
PwPPA Version 1.4: February 3, 2023

PART I. INTRODUCTION AND REVIEW OF COMMUNICATION PARTICIPATION ITEM BANK (CPIB) SHORT FORM

Read to Participant:

The purpose of this interview is to get your feedback on a brief questionnaire called the Communication Participation Item Bank (CPIB). We're interested in your candid thoughts and opinions, there are no right or wrong answers. The interview is being recorded so that I don't miss any of your comments. I want to emphasize that your comments are strictly confidential. No names will be used in any reports or publications. All names and other identifying information will be removed from the transcripts.

During this interview it is very important that we obtain feedback on the questionnaire from individuals with PPA in their own words. However, given the nature of PPA, we are inviting communication partners to listen in and be available to assist as needed. [PPA NAME], if you are experiencing any confusion or would like support from [CP NAME], please feel free to ask [CP NAME] for help. [CP NAME], for this study it is very important we hear directly from the participant and we want to be sure to provide enough time to consider the question and answer, so please wait to be asked by [CP NAME] before assisting with responses. You will have an opportunity to share your perspectives in your upcoming one-on-one interview.

Do you have any questions before we begin?

[TURN ON RECORDER; STATE PARTICIPANT ID AND DATE]

[Interviewer: Participant completes the CPIB questionnaire. Keep the recorder ON as s/he completes the instrument to capture any questions the participant may have about how to complete the questionnaire, or any confusion expressed by the participant].

You just completed a questionnaire called the Communication Participation Item Bank (CPIB). We would appreciate your feedback regarding this questionnaire to understand whether the questionnaire is clear and is relevant to your experiences with PPA. Before discussing your thoughts of each of the items, I'd first like to ask you some general questions about the questionnaire overall.

9. Did you think about a specific period of time, when answering the questions? ___ No (0)
___ Yes (1)

If No --> **How did you decide how to answer these questions?**

If Yes --> **What period of time did you think about when answering the questions?** (e.g., Did you think about your experiences today, last week, last year?)

10. Tell me what the instructions asked you to do? (e.g., If you were going to tell me how to complete the survey, how would you explain it to me?)

11. Were the instructions clear? ___ No (0)
___ Yes (1)

If No →**Why?**

12. Each question is listed in its own box. Was it good that the questions were in their own boxes? ___ No (0)
___ Yes (1)

If No →**Why?**

13. The answer choices are shown on lines. Did the lines make sense to you? ___ No (0)
___ Yes (1)

If No →**Why?**

14. Where did you mark your answer on the questionnaire? (e.g., Did you have questions about how to mark your answer?)

15. Was the format of the questionnaire clear to you? ___ No (0)
___ Yes (1)

If No → **Is there a way we could reformat the questionnaire to make it more clear?**

16. These questions ask you to respond using "Not at all, A little, Quite a bit, and Very much". Do these response options reflect the range of your experiences communicating in different situations/environments?

___ No (0)
___ Yes (1)

If No →**Why?**

Optional probes:

- How did you decide which answer to choose? (e.g., average your experiences, think of your best/worst experience, or more recent experience)
- Was there ever a time you wanted to answer halfway in between? If yes→**Why?**

17. Was it easy to answer using those options?

___ No (0)
___ Yes (1)

If No →**Why?**

18. Next, I'm going to ask you a series of questions about each of the items on the questionnaire. <u>For this set of questions, I am going to put the main interview questions on the screen so you can follow along. But I might ask additional follow-up questions that are not on the screen. Please let me know if you are unable to see the questions or if they are not advancing.</u> [*Interviewer: If participant appears confused*

when responding to questionnaire or when discussing specific items (e.g., struggles/asks questions about an item, changes his/her answer after discussing an item) mark the checkbox in Column 1 for that item and take notes on the issue.]

Item<br><br>[ASK FOR RESPONSE]	**a. What did you think about when you answered the question?**<br>*(i.e., Why did you choose [RESPONSE]?)*<br><br>*Optional probe: specific time/experience*	**b. Was the meaning of the question clear?**<br>Yes/No<br>**<u>If no</u>, what was unclear?**<br><br>**c. *Optional:***<br>***What does the word / phrase _____ mean?**	DO NOT ASK IF ANSWERED 3 "NOT AT ALL".<br><br>**d. For this question, how much would your answer need to change to have a meaningful impact on your life?**<br>*(e.g., If your score improved 1 point from __ to __, would that be meaningful to your life? Please explain.)*	**e. Is this question relevant to your experiences with PPA?** (e.g., situation where PPA interferes with __)<br>Yes/No<br>**<u>If no, Why?</u>**<br><br>**f. Should we make changes to this question?**<br>Yes/No<br>**If yes, what changes?**
1. Does your condition interfere with talking with people you know?<br><br>☐	**a.**	**b.**<br><br>**c.** *interfere with*	**d.**	**e.**<br><br>**f.**
2. Does your condition interfere with communicating when you need to say something quickly?<br><br>☐	**a.**	**b.**<br><br>**c.** *N/A*	**d.**	**e.**<br><br>**f.**

Item<br><br>[ASK FOR RESPONSE]	**a. What did you think about when you answered the question?**<br>*(i.e., Why did you choose [RESPONSE]?)*<br><br>*Optional probe: specific time/experience*	**b. Was the meaning of the question clear?**<br>Yes/No<br>**If no, what was unclear?**<br><br>**c. *Optional:***<br>***What does the word / phrase _____ mean?**	**DO NOT ASK IF ANSWERED 3 "NOT AT ALL".**<br><br>**d. For this question, how much would your answer need to change to have a meaningful impact on your life?**<br>*(e.g., If your score improved 1 point from __ to __, would that be meaningful to your life? Please explain.)*	**e. Is this question relevant to your experiences with PPA?** (e.g., situation where PPA interferes with __)<br>Yes/No<br>**If no, Why?**<br><br>**f. Should we make changes to this question?**<br>Yes/No<br>**If yes, what changes?**
3. Does your condition interfere with talking with people you do NOT know?<br><br>☐	**a.**	**b.**<br><br>**c.** *N/A*	**d.**	**e.**<br><br>**f.**
4. Does your condition interfere with communicating when you are out in your community (e.g., errands, appointments)?<br><br>☐	**a.**	**b.**<br><br>**c.** *N/A*	**d.**	**e.**<br><br>**f.**

Item<br><br>**[ASK FOR RESPONSE]**	**a. What did you think about when you answered the question?**<br>*(i.e., Why did you choose [RESPONSE]?)*<br><br>*Optional probe: specific time/experience*	**b. Was the meaning of the question clear?**<br>Yes/No<br>**If no, what was unclear?**<br><br>**c. *Optional:***<br>***What does the word / phrase _____ mean?**	**DO NOT ASK IF ANSWERED 3 "NOT AT ALL".**<br><br>**d. For this question, how much would your answer need to change to have a meaningful impact on your life?**<br>*(e.g., If your score improved 1 point from __ to __, would that be meaningful to your life? Please explain.)*	**e. Is this question relevant to your experiences with PPA?** (e.g., situation where PPA interferes with __)<br>Yes/No<br>**If no, Why?**<br><br>**f. Should we make changes to this question?**<br>Yes/No<br>**If yes, what changes?**
5. Does your condition interfere with asking questions in a conversation?<br><br>☐	**a.**	**b.**<br><br>**c.** *N/A*	**d.**	**e.**<br><br>**f.**
6. Does your condition interfere with communicating in a small group of people?<br><br>☐	**a.**	**b.**<br><br>**c.** *N/A*	**d.**	**e.**<br><br>**f.**

Item<br><br>[ASK FOR RESPONSE]	**a. What did you think about when you answered the question?**<br>*(i.e., Why did you choose [RESPONSE]?)*<br><br>*Optional probe: specific time/experience*	**b. Was the meaning of the question clear?**<br>Yes/No<br>**If no, what was unclear?**<br><br>**c. *Optional:***<br>***What does the word / phrase _____ mean?**	**DO NOT ASK IF ANSWERED 3 "NOT AT ALL".**<br><br>**d. For this question, how much would your answer need to change to have a meaningful impact on your life?**<br>*(e.g., If your score improved 1 point from __ to __, would that be meaningful to your life? Please explain.)*	**e. Is this question relevant to your experiences with PPA?** (e.g., situation where PPA interferes with __)<br>Yes/No<br>**If no, Why?**<br><br>**f. Should we make changes to this question?**<br>Yes/No<br>**If yes, what changes?**
7. Does your condition interfere with having a long conversation with someone you know about a book, movie, show, or sports event?<br><br>☐	**a.**	**b.**<br><br>**c.** *long conversation*	**d.**	**e.**<br><br>**f.**
8. Does your condition interfere with giving someone DETAILED information?<br><br>☐	**a.**	**b.**<br><br>**c.** *detailed information*	**d.**	**e.**<br><br>**f.**

Item<br><br>**[ASK FOR RESPONSE]**	**a. What did you think about when you answered the question?**<br>*(i.e., Why did you choose [RESPONSE]?)*<br><br>*Optional probe: specific time/experience*	**b. Was the meaning of the question clear?**<br>Yes/No<br>**If no, what was unclear?**<br><br>**c. *Optional:***<br>***What does the word / phrase _____ mean?**	**DO NOT ASK IF ANSWERED 3 "NOT AT ALL".**<br><br>**d. For this question, how much would your answer need to change to have a meaningful impact on your life?**<br>*(e.g., If your score improved 1 point from __ to __, would that be meaningful to your life? Please explain.)*	**e. Is this question relevant to your experiences with PPA?** (e.g., situation where PPA interferes with __)<br>Yes/No<br>**If no, Why?**<br><br>**f. Should we make changes to this question?**<br>Yes/No<br>**If yes, what changes?**
9. Does your condition interfere with getting your turn in a fast-moving conversation?<br><br>☐	**a.**	**b.**<br><br>**c.** *getting your turn*	**d.**	**e.**<br><br>**f.**
10. Does your condition interfere with trying to persuade a friend or family member to see a different point of view?<br><br>☐	**a.**	**b.**<br><br>**c.** *to persuade*	**d.**	**e.**<br><br>**f.**

PART II. SUMMARY QUESTIONS

Next, I'm going to ask you about these questions.

6. **What were these questions asking you about?**

7. **Which questions were difficult to answer?** *For each question identified, ask:* **What made it difficult to answer?** [*Interviewer takes note of questions and reasons.*]

8. **Please take a moment to look over the questions again. Do these questions capture your experiences with PPA?**

   ___ No (0)

   ___ Yes (1)

   If No →**Why?**

Before we wrap up, I'd like you to take a moment and look over the questions again. Can you tell me about any other important communication situations or environments that are missing from the questionnaire? [*Interviewer takes note of questions/situations below and probes for details and examples of each]*

___ No (0)

___ Yes (1)

4a. List all missing communication experiences/situations provided by participant	***4b. Rating***

4b. *[Interviewer ask if completed Q4a]:* **Next, I would like you to think about how important each of these communication experiences/situations are to your ability to participate in your life. On a 0-10 scale, please rate each of these communication experiences/situations on its importance your ability to participate in your life. For the ratings, 0 is Not at all important and 10 is Extremely important.** *[Interviewer: Read each of the experiences/situations listed under 4a and ask them to rate it from 0-10. Indicate their rating next to the concern in Column **4b** above. Encourage PT to select a whole number]*

4c. *For the situations/experiences rated the highest (typically 1-2 concerns, but possibly more), ask the participant:* **You rated _______ as one of the most important communication situations/experiences to your ability to participate in your life. Why is that situation/experience most important to you?**

5. **Is there anything else that you would like to suggest that would help us to improve these questions or the questionnaire?**

PART III. CLOSING

Thank you for your sharing your feedback and experiences with me today. Your input is very important in helping us to evaluate the appropriateness of this questionnaire for individuals with PPA.

[TURN OFF RECORDER].

[Mailed Gift Card] **Ollie will send you a $50 gift card in the mail. Would you like her to send the mailing address we used to send the interview materials?** [*Interviewer writes name and mailing address below if different.*] **When you receive your $50 gift card, please sign and date the enclosed form and send it back to us in the stamped and addressed envelope provided. We need the form to process the paperwork.**

Name:

Mailing Address:

[Virtual Gift Card] **We will send you a $50 virtual gift card. What email address would you like the gift card to be sent to? You will receive an email within 72 hours from Ollie Fegter, part of the Communication Bridge study team] with instructions on how to activate and access your virtual gift card.**

>>For interviewer: Even if you have an email address on file and participant says "the same email that you sent the consent to," be sure to repeat the email address back to them to confirm.

Email Address:
